# Orlistat after initial dietary/behavioural treatment: changes in body weight and dietary maintenance in subjects with sleep related breathing disorders

**DOI:** 10.1186/1475-2891-10-21

**Published:** 2011-03-08

**Authors:** Mette Svendsen, Serena Tonstad

**Affiliations:** 1 Preventive Cardiology, Oslo University Hospital, Ullevål Hospital, N-0407 Oslo, Norway

## Abstract

**Background:**

Sleep related breathing disorders (SRBD) are associated with increased morbidity and mortality and weight loss is recommended to overweight or obese patients with SRBD. However, maintenance of weight loss is difficult to achieve and strategies for weight loss maintenance is needed. Orlistat is a pharmacological agent that reduces the intestinal absorption of fat and may favour long-term weight maintenance.

**Objective:**

To examine the change in body weight and dietary intake during a 1-year treatment with orlistat after an initial weight loss in obese subjects with SRBD. Furthermore, to explore the dietary determinants of weight maintenance during treatment with orlistat.

**Methods:**

Men and women with SRBD aged 32-62 years (n = 63) participated in a 3-month dietary intervention to increase intake of vegetables and fruit. After an initial weight loss of 3.4 kg they achieved a mean body mass index of 34.3 ± 4.7 kg/m2. Subsequently they were treated with orlistat for 1 year. During this year, dietary and behavioural interventions to attain weight loss were provided in the course of 14 group sessions. Dietary intake, energy density and food choices were assessed with a food frequency questionnaire before and after orlistat treatment.

**Results:**

With orlistat, body weight decreased by a mean of 3.5 kg (95% CI 1.5, 5.5). The dietary E% from saturated fat, intake of fatty dairy products and energy density increased after 1 year while intakes of oils, fish and vegetables decreased (all P < 0.05). After multivariate adjustments, weight loss was associated with E% protein (R2_adj _= 0.19 [95% CI 0.10, 0.46]), and inversely associated with E% saturated fat (R2_adj _= 0.20 [95% CI 0.12, 0.47]) and fatty dairy products (R2_adj _= 0.23 [95% CI 0.12, 0.49]).

**Conclusions:**

Orlistat induced further weight loss, but dietary compliance declined with time. Increasing dietary protein and restricting saturated fat and fatty dairy products may facilitate weight loss with orlistat.

## Background

Sleep related breathing disorders (SRBD), a condition characterized by repeated episodes of apnoea and hypo-apnoea during sleep, is associated with increased morbidity and mortality [1]. The prevalence of SRBD increases with increasing body mass index (BMI) and weight loss is universally recommended to overweight or obese patients with SRBD [2]. Recently, randomized, long-term intervention studies have shown clinical relevant improvements in the apnoea and hypo-apnoea index with weight loss of about 10 kg [3,4].

In most weight loss programs an increased intake of vegetables and fruit is recommended in energy reduced diets. Vegetables and fruit have relatively high water content that adds weight but not energy to the diet and these food items decrease the energy density of the diet. Indeed, dietary advice to decrease the energy density of the diet has been suggested as a strategy for reducing energy intake and body weight [5]. The energy density of the diet is also influenced by the intake of fat because fat has a higher energy density (38 kJ/g) than carbohydrate or protein (17 kJ/g) [6,7]. Thus, fat-restricted diets would decrease energy density.

Orlistat is a pancreatic lipase inhibitor that reduces the intestinal absorption of fat [8]. Gastrointestinal symptoms including oily spotting, faecal incontinency and flatulence may occur if the dietary intake of fat is higher than recommended. Thus, orlistat may have a policing effect on the intake of fatty foods. However, randomized clinical trials have shown that the intake of fat in subjects taking orlistat or placebo does not differ substantially [9,10]. An increased intake of fat during long-term treatment with orlistat has also been shown [11].

We previously reported the results of a 3-month group-based dietary and behavioural program in subjects with SRBD [12]. The intervention group doubled the intake of vegetables and fruit, reduced energy from total fat and saturated fat and reduced dietary energy density. Following the trial, subjects were offered continued dietary and behavioural counselling and orlistat treatment for 1 year. In this study we examined dietary intake, energy density, food choices and dietary determinants of weight change during 1 year of orlistat treatment in obese subjects with SRBD. To our knowledge, the impact of orlistat on energy density of the diet has not been studied previously.

## Materials and methods

### Subjects

The subjects were referred from the Ear, Nose and Throat Department or primary care physicians to Preventive Cardiology at the Oslo University Hospital for weight reduction. The diagnosis of SRBD was verified during polysomnography in a sleep laboratory mostly done at the hospital. In brief, inclusion criteria were men and women with SRBD aged 21 to 72 years with a BMI ≥ 27 kg/m2. Exclusion criteria were drug or alcohol abuse or lack of motivation, major non-cardiac disease expected to reduce life expectancy or interfere with the study and the use of appetite suppressants or weight reducing medication within the last 3 months. The Ethical Committee (region 1) in Norway approved the protocol and all participants provided written informed consent.

### Study design

Immediately after completing the 3-month weight loss program [12] participants in the intervention group (n = 63) were offered orlistat free of charge in addition to continued dietary and behavioural counselling. The participants were instructed to take orlistat capsules (120 mg) 3 times daily at each main meal. At monthly visits body weight and waist circumference were measured, adverse events were reported and orlistat capsules were delivered.

Behavioural treatment consisted of 14 group sessions lasting 90 minutes and scheduled at monthly intervals except for the first four sessions that were scheduled every second week. Subjects were asked to follow a diet consisting of < 30% of energy from total fat and < 10% of energy from saturated fat. They were asked to consume low fat dairy products and lean meat or chicken instead of minced meat and sausages, and to reduce intake of cakes, biscuits, ice cream, chocolate and snacks. To increase the palatability of the diet, a small amount of unsaturated fat from fatty fish and plant sources was recommended. Food exhibits demonstrated the amount of fat in various foods. Subjects were taught to read food labels and focus on the fat content. They were instructed in planning for social eating and eating at restaurants. They were given homework to assess their eating behaviour and to plan specific strategies to avoid pitfalls. The behavioural program emphasized goal setting, stimulus control and cognitive restructuring [13].

### Dietary assessments

A registered dietician (MS) conducted a dietary interview based on a food frequency questionnaire (FFQ) before and after 1 year of treatment with orlistat. The FFQ was designed to assess dietary intake during the last 3 months and has been described in detail elsewhere [14]. In short, the questionnaire elicited frequencies and consumption of 174 food items. Portion sizes were estimated with the use of a photographic atlas, photographs of food items and standardized measurement units. The FFQ was coded manually for the calculation of total energy, energy density, energy, nutrients and food items using a software program (Mat på data 3.0, 1996) based on the Norwegian food composition table [15]. Energy density was calculated for the entire diet minus drinks (coffee, tea, milk, juice, soft drinks and alcoholic beverages).

### Anthropometry

Subjects were weighed (in underwear) with a digital weight (Seca, Germany) to the nearest 0.1 kg before start of treatment with orlistat and after 1 year. Height was measured with a standardized wall measuring stick scale to the nearest 0.5 cm. Waist circumference was measured at the umbilicus with the subject unclothed and in the standing position.

### Statistical analysis

Subjects were divided into attendance groups. The subjects in the high attendance group attended more group sessions than the mean which was 9.34 sessions and the subjects in the low attendance group attended ≤ 9.34 sessions. For the high attendance group the attendance rate was > 66.7% and for the low attendance group the attendance rate was ≤ 66.7%.

The results are presented as means (SD or 95% confidence intervals CI) or median and 25th, 75th percentiles as appropriate. Mean differences within and between groups were tested with paired and unpaired t-tests, respectively. Differences in skewed variables between groups were tested with the Mann-Whitney two-sample rank test. Pearson or Spearman correlations were used for normally distributed or skewed variables, respectively, to explore the associations between single, continuous variables. Multiple regression analyses were conducted to identify adjusted associations between dietary variables and weight loss during treatment with orlistat. The tests were considered statistically significant at P < 0.05. Analyses were performed using the Stat View 5.0.1 software (Abacus concepts, Berkeley, California, USA).

## Results

The 48 men and 15 women in the study were between 32 and 62 years of age and had a mean BMI of 34.6 ± 4.7 kg/m2. Five men did not attend the behavioural sessions and are not included in the analyses. Their baseline characteristics (age, anthropometric measurements, established cardiovascular disease or diabetes mellitus, pharmacological therapy and treatment with CPAP) did not differ from men that followed the group sessions (data not shown).

### Change in dietary intake

Table 1 shows the dietary intake data at 1 year and changes from the start of orlistat. Reported energy density and energy percent (E%) from saturated fat was increased and E% from monounsaturated fat, carbohydrate and grams of fibre was reduced. Energy density increased more in the low attendance group than in the high attendance group. Intakes of fatty dairy products (butter, cream and fatty cheeses) were increased and the intake of oil, fish and vegetables were decreased in the groups as a whole (Table 2).

**Table 1:** Dietary intake after 1 year of treatment with orlistat

	All participants (n = 63)	High attendance group (n = 31)	Low attendance group (n = 32)	P*
**Energy intake (kJ)**				
1 year	9229 ± 2304†	8747 ± 2033	9696 ± 2483	
Change‡	178 [-366, 722] §	-57 ± 1618	406 ± 2661	0.409
**Energy density (kJ/g)**				
1 year	5.3 ± 1.5	4.7 ± 1.2	5.9 ± 1.5	
Change	0.5 [0.2, 0.7]	0.1 ± 0.9	0.8 ± 1.0	0.008
**Fat (E%)**				
1 year	29.7 ± 7.3	27.3 ± 6.5	32.0 ± 7.4	
Change	1.7 [-0.2, 3.6]	0.2 ± 6.6	3.2 ± 8.5	0.117
**Saturated fat (E%)**				
1 year	10.1 ± 3.7	8.6 ± 2.4	11.5 ± 4.1	
Change	1.2 [0.4, 2.0]	0.7 ± 1.7	1.7 ± 4.2	0.201
**Monounsaturated fat (E%)**				
1 year	10.1 ± 3.7	8.6 ± 2.4	11.5 ± 4.1	
Change	-1.3 [-2.2, -0.3]	-2.6 ± 2.9	0 ± 4.5	0.009
**Polyunsaturated fat (E%)**				
1 year	5.4 ± 1.7	5.3 ± 2.1	5.4 ± 1.3	
Change	0 [-0.5, 0.5]	-0.3 ± 2.2	0.3 ± 1.4	0.169
**Protein (E%)**				
1 year	17.4 ± 2.7	18.3 ± 2.5	16.5 ± 2.6	
Change	0 [-0.6, 0.8]	0.8 ± 3.3	0.3 ± 1.4	0.051
**Carbohydrate (E%)**				
1 year	47.7 ± 6.5	48.7 ± 6.3	46.7 ± 6.5	
Change	-1.9 [-3.7, -0.2]	-0.4 ± 5.9	-3.4 ± 1.4	0.089
**Fiber (g/MJ)**				
1 year	3.9 ± 1.4	4.3 ± 1.5	3.5 ± 1.2	
Change	-0.3 [-0.6, -0.1]	-0.1 ± 1.2	-0.5 ± 0.8	0.143

* Comparison of changes between the high and low attendance group (unpaired t-test). The subjects in the high attendance group attended more group sessions than the mean which was 9.34 sessions and the subjects in the low attendance group attended = 9.34 sessions.

† Mean ± SD ‡ Changes are calculated as the difference between 1 year and the start of orlistat. § Mean; 95% CI in brackets.

**Table 2:** Daily intake of food after 1 year of treatment with orlistat in all participants (n = 63)

Food items	Mean ± SD	Change* [95% CI]	P†
Vegetables, g	448 ± 235	-49 [-103, 5]	0.050
Fruit‡, g	424 ± 265	-42 [-120, 37]	0.122
Bread, g	162 ± 75	2 [-15, 19]	0.820
Potatoes, rice and pasta, g	155 ± 88	-4 [-28, 20]	0.421
Poultry and lean meat, g	110 ± 56	6 [-9, 20]	0.661
Fish, g	63 ± 51	-10 [-19, -1]	0.027
Fatty meat‡, g	46 ± 42	7 [-4, 18]	0.154
Sweets, cookies and desserts, g	43 ± 43	5 [-5, 15]	0.457
Butter, cream and fatty cheeses, g	32 ± 36	11 [1, 21]	0.018
Oil‡, g	16 ± 11	-4 [-7, 0]	0.160

* Changes were calculated as the difference between intake at 1 year and start of the treatment with orlistat. † Comparisons between the intake of food at start of the treatment with orlistat and after 1 year (the Wilcoxon signed rank test). ‡ Fatty meat includes minced meat, sausages, pork chops, salami; fruit includes orange juice, berries and berry jam; oil includes original and fat reduced soft margarine, mayonnaise and salad-dressings.

### Change in anthropometrics

Before orlistat was started the mean BMI was 34.3 ± 4.5 kg/m2 (range 25.9-45.6 kg/m2), and a mean waist circumference of 113.3 ± 13.8 cm (range 86-142 cm). After 1 year the mean weight reduction was -3.5 ± 8.1 kg [95% CI -1.5, -5.5 kg]. The high attendance group achieved better weight reduction than the low attendance group (mean, -6.2 ± 6.6 kg] versus -0.9 ± 8.7 kg, P = 0.008). Likewise, the high attendance group reduced waist circumference more than the low attendance group (mean, -4.4 ± 6.3 cm versus -1.9 ± 7.3 cm, P = 0.001). Change in weight was inversely correlated to attendance rate (r = -0.34, [95% CI -0.10, -0.55], P = 0.056).

### Change in dietary intake as predictors for weight loss

Table 3 shows the multiple regression analyses of change in E% of protein, E% of saturated fat and fatty dairy products as predictors for weight loss after adjustments for intake at baseline, attendance rate, age, gender and BMI at baseline. After adjustments, change in E% of protein (R2_adj _= 0.19 [95% CI 0.01, 0.46]), E% of saturated fat (R2_adj _= 0.20 [95% CI 0.12, 0.47] and intake of fatty dairy products (R2_adj _= 0.23 [95% CI 0.12, 0.49]) predicted weight loss. Changes in energy density, E% monounsaturated fat, carbohydrates, fibre, vegetables, fish and oil were not associated with change in weight (all P values >0.1).

**Table 3:** Multiple regression analysis of change in dietary variables as predictors for change in weight (n = 63)

	Standardised regression coefficients	P
**Changes in energy percent saturated fat**	0.264	0.038
Energy percent saturated fat before weight loss	0.240	0.063
Attendance rate	-0.340	0.006
Age	0.257	0.034
Gender	0.088	0.463
BMI before weight loss	-0.224	0.082
**Change in energy percent protein**	-0.276	0.028
Energy percent protein before weight loss	0.186	0.113
Attendance rate	-0.360	0.004
Age	0.146	0.222
Gender	0.089	0.460
BMI before weight loss	-0.089	0.446
**Change in intake of cream, butter and fatty cheeses**	0.296	0.011
Cream, butter and fatty cheeses before weight loss	0.224	0.063
Attendance rate	-0.371	0.002
Age	0.254	0.032
Gender	0.019	0.869
BMI before weight loss	-0.122	0.303

## Discussion

This was a study of a 1-year group-based dietary/behavioural program combined with orlistat following 3 months of dietary counselling in patients with SRBD. Our main findings were that the program was associated with reduced body weight, but compliance to dietary recommendations deteriorated somewhat during treatment. Dietary energy density, E% from saturated fat and intakes of fatty dairy products increased during the 1-year period. In the multiple regression analyses, further loss of body weight was associated with increased intake of protein and lower E% saturated fat and fatty dairy product intakes. These variables explained almost 20% of the variation in weight reduction. The strengths of our study are that we have follow-up data on all subjects included in the dietary/behavioural program and comprehensive dietary data. A limitation is the lack of a control group however the study was designed to demonstrate the results of treatment in a usual clinical setting.

Despite behavioural and pharmacological support, compliance with the diet worsened in all subjects during the year of treatment. Deterioration of dietary compliance after an initial intervention during long-time follow-up is common [16]. Our data suggest that even treatment with a pharmacological aid as orlistat and moderately intense behavioural support does not prevent this deterioration. However we did observe that the group with high attendance showed better dietary maintenance than the low attendance group.

The increase in the intake of fat during 1 year is surprising in light of the mechanisms of action of orlistat which may lead to gastrointestinal problems if dietary fat is not restricted. Gastrointestinal symptoms are thought to occur with a dietary intake of fat >30% of total energy [17], but individual tolerability may vary. Thus, subjects may increase their intake of fatty dairy products without experiencing gastrointestinal problems. It has also been speculated that subjects treated with orlistat learn to titrate their dietary intake of fatty foods [11]. Another possibility is that some subjects did not use the medication as prescribed.

We found that energy density increased during the year of follow-up however, change in energy density was not associated with weight loss. On the other hand, the high attendance group maintained the reduction in energy density during treatment with orlistat more successfully than the low attendance group. A reduced energy density has been associated with weight loss in two previous studies [18,19]. The PREMIER study showed improved weight loss in subjects with the largest reduction in energy density compared to the subjects with the lowest reduction [18]. The study by Ello-Martin et al. showed that subjects who reduced fat intake with a concomitant increase in vegetables and fruit lost more in weight and reported less hunger compared to subjects that only reduced their fat intake [19] supporting the notion that energy density is a determinant of weight loss.

We found that increased protein intake was associated with improved weight loss, in line with previous data [20-22]. Dietary protein is associated with increased energy expenditure and less hunger [22]. We speculate that less hunger may have particular importance for subjects with SRBD since reduced sleep quality may interfere with appetite [23].

The amount of weight loss during the year of treatment with orlistat was about 5%. However, the subjects had already lost about 3 kg of weight prior to the initiation of orlistat. This total change is in accordance with the weight reduction achieved in a sibutramine assisted weight loss program in obese males with SRBD [24]. In this study, favourable changes in the frequency of disordered breathing events and cardio-metabolic risk factors were shown concomitant with the loss in weight [24,25]. A limitation of our study is that we did not measure the apnoea - hypo apnoea index after weight reduction. However, most of the participants used continuous positive airway pressure for symptomatic treatment of their SRBD before weight loss was initiated [12].

## Conclusions

This study showed that treatment with orlistat for 1 year in a group-based behavioural program was associated with further weight reduction after an initial weight loss. However, dietary energy density, and intakes of saturated fat and fatty dairy products increased. Increasing dietary protein and restricting fatty dairy products may improve success with orlistat in obese subjects with SRBD.

## Competing interests

The authors declare that they have no competing interests.

## Authors' contributions

ST and MS contributed to the development of the protocol. MS performed all the dietary assessments and was responsible for data analysis, interpretation and manuscript preparation. ST was the principal investigator of the study, performed medical assessments, assisted with manuscript preparation and critically reviewed the manuscript. Both authors have read and approved the final version of the manuscript.
